# Comparison of Initial Clinical Presentations between Primary Hyperparathyroidism Patients from New Brunswick and Changsha

**DOI:** 10.1155/2018/6282687

**Published:** 2018-09-18

**Authors:** Lingqiong Meng, Shuying Liu, Aseel Al-Dayyeni, Zhifeng Sheng, Zhiguang Zhou, Xiangbing Wang

**Affiliations:** ^1^Division of Endocrinology and Metabolism, Rutgers-RWJMS, New Brunswick, NJ 08901, USA; ^2^Graduate School of Biomedical Science, Rutgers University, Piscataway, NJ 08854, USA; ^3^Department of Metabolism and Endocrinology, The Second Xiangya Hospital, Central South University, Changsha, Hunan 410011, China; ^4^Physiology and Integrative Biology, Rutgers University, New Brunswick, NJ 08901, USA; ^5^National Clinical Research Center for Metabolic Diseases, The Second Xiangya Hospital, Central South University, Changsha, Hunan 410011, China; ^6^Institution of Metabolism and Endocrinology, The Second Xiangya Hospital, Central South University, Changsha, Hunan 410011, China

## Abstract

**Purpose:**

To compare the initial clinical features, laboratory values, and bone mineral density among patients with primary hyperparathyroidism (PHPT) in Changsha (China) and New Brunswick (USA).

**Methods:**

In this retrospective study, we reviewed 169 PHPT patients who presented at Robert Wood Johnson University Hospital and 133 PHPT patients who presented at the Second Xiangya Hospital of Central South University in the same time period. The following characteristics were compared between the groups: age, gender, BMI, serum calcium, alkaline phosphatase (AKP), albumin, intact PTH (iPTH), 25-hydroxyvitamin D (25 (OH) D), fasting blood glucose levels, and bone mineral density (BMD). All these parameters were also compared according to gender and menopausal status. iPTH associations were also assessed along with several other parameters.

**Results:**

PHPT patients from Changsha had higher serum calcium, iPTH, and AKP levels but lower 25 (OH) D levels than the patients from New Brunswick (*p* < 0.05). Patients in Changsha had lower *T*-scores and *Z*-scores in both the lumbar spine and hip regions than those in New Brunswick (*p* < 0.05). Patients in New Brunswick had lower percentages of parathyroid adenoma and kidney stones. Serum iPTH level was positively correlated with serum calcium and serum AKP levels in both Changsha and New Brunswick (*p* < 0.05).

**Conclusions:**

There are distinct biochemical and clinical differences between patients with PHPT in China and the United States. Our study revealed that Asian PHPT patients from Changsha presented more severe PHPT profiles, lower bone mineral density, and higher incidence of renal stones.

## 1. Introduction

The use of multichannel chemistry screening tests in the United States and other Western countries since the 1970s has made primary hyperparathyroidism (PHPT) a more commonly recognized endocrine disease, and it has shifted from being a classically symptomatic disorder to an asymptomatic one [1, 2]. Nowadays, most patients with PHPT in the US are asymptomatic as compared with their counterparts in developing Asian countries, such as India and China, who are still characterized with distinct clinical manifestations and severe biochemical markers [3]. Currently, few data are available on PHPT comparisons between China and the USA.

Although a recent study showed a mild increase in asymptomatic PHPT patients in Shanghai, there were still major differences in clinical presentations between Shanghai (China) and New York City (USA). The data suggested that geographical locations, ethnicities, and other factors may contribute to the overt differences in clinical and biochemical manifestations [4]. However, the PHPT patients from the two cities were from different time periods: Shanghai (2001–2010) and New York (2009–2013). The PHPT profiles of China might have changed, as the use of multichannel chemistry tests and ultrasound examination of the neck has recently become widely available in China. We therefore compared the initial presentation of patients with PHPT from Changsha, China, and New Brunswick, USA, in the same time period. The comparison of clinical characteristics of PHPT from different geographical locations or ethnicities from the same time periods may help clinicians do a better job diagnosing and managing PHPT patients from different ethnic backgrounds. Our current study may also provide clues for understanding the role of genetic, environmental, and nutritional factors in the development of PHPT.

## 2. Methods

This is a chart review and retrospective study. Medical records of PHPT patients at the Divisions of Endocrinology and General Surgery at Robert Wood Johnson University Hospital (New Brunswick, NJ) and the Division of Endocrinology at the Second Xiangya Hospital of Central South University (Changsha, China) from 2010 to 2016 were reviewed separately and identified. The data collection was finished by trained endocrinologists in both countries. The initial diagnoses of primary hyperparathyroidism were diagnosed through (1) confirmed hypercalcemia (calcium > 10.4 mg/dL) and (2) elevated intact PTH levels (iPTH > 65 pg/mL). Patients diagnosed with multiple endocrine neoplasm (MEN) and parathyroid carcinoma were excluded. The protocols were approved by the Institutional Review Boards of Rutgers University (New Brunswick, USA) and the Second Xiangya Hospital (Changsha, China).

Age, gender, menopausal status, BMI, level of serum calcium, iPTH, 25 (OH) D, albumin, AKP, creatinine, and fasting blood glucose levels were recorded. Albumin-corrected serum calcium was calculated by the following formula: corrected calcium = serum calcium + 0.8 × (4 − serum albumin). All patients from two cities were queried for the history of kidney stones. Past medical history of renal stones was expressed as a percentage. For patients who underwent parathyroidectomy (158 and 92 PHPT patients in New Brunswick and Changsha, respectively), adenoma or hyperplasia was determined by a pathologist according to pathological features of parathyroid glands [5] and confirmed by another pathologist. We also collected lumbar spine and total hip BMD using dual-energy X-ray absorptiometry (DXA) with the GE Lunar Prodigy Advance (CV ≤ 1%, *T*-score and *Z*-score) in patients from both cities. iPTH associations were also assessed along with several other parameters.

Serum calcium, AKP, serum creatinine, serum albumin, and fasting blood glucose were measured by cobas e analyzers using standard enzymatic and colorimetric assay protocols in both cities. Serum iPTH levels were measured by cobas e analyzers with the normal range 15–65 pg/mL, and serum 25 (OH) D levels were assessed using cobas automated radioimmunoassay analyzers with the normal range 30–80 ng/mL in New Brunswick. Serum iPTH levels were measured by Elecsys analyzers with the normal range 14–63 pg/mL, and serum 25 (OH) D levels were measured by Siemens analyzers with the normal range 30–100 ng/mL in Changsha.

### 2.1. Statistical Analysis

The results that were normally distributed were expressed as mean ± SD, while those without normal distribution were expressed as median (range). Normal distributions between groups were compared with the Student *t*-test. Nonnormal distributions between groups were compared with the Wilcoxon rank sum test. The prevalence rates between groups were compared using Chi-square tests for significance. Correlation coefficients, linear regression, and logistic regression were used to assess relationships. Two-sided *p* values of <0.05 were selected as the significance level.

## 3. Results

We included 169 PHPT patients (130 females and 39 males) from Robert Wood Johnson University Hospital and 133 PHPT patients (87 females and 44 males) from the Second Xiangya Hospital. Two PHPT patients from Changsha were diagnosed with parathyroid cancer, while no parathyroid cancer was found in the New Brunswick sample. We excluded parathyroid cancer patients for better comparison. PHPT patients from New Jersey included 138 Caucasian, 13 Black, 10 Hispanic, and 8 Asian patients.  Table 1 shows the clinical characteristics and biochemical values of PHPT patients from both cities. Compared to PHPT patients from New Brunswick, PHPT patients from Changsha were younger, with lower BMI, higher serum calcium, iPTH, and AKP levels, and lower 25 (OH) D levels (*p* < 0.05). Fasting glucose was also higher in New Brunswick PHPT patients than in those patients from Changsha (*p* < 0.05, Table 1). Compared to PHPT patients from New Brunswick, PHPT patients from Changsha had more kidney stones at the initial evaluation (*p* < 0.001, Table 1). For patients that had parathyroidectomy, pathology reports showed that 92% of PHPT patients from Changsha had parathyroid adenoma while only 48% of PHPT patients from New Brunswick had parathyroid adenoma (Table 1, *p* < 0.001).

There are 34% male PHPT patients and 25% premenopausal female PHPT patients from Changsha while there are only 25% male PHPT patients and 17% premenopausal PHPT patients from New Brunswick (Table 2). Males and premenopausal females have higher calcium and PTH levels but lower 25 (OH) D levels than the postmenopausal females in both cities. Compared to PHPT patients from New Brunswick, all PHPT patients (male and pre- or postmenopausal female) from Changsha had higher serum calcium, iPTH, and AKP levels and lower 25 (OH) D levels. All PHPT patients from Changsha also had lower *T*-scores in the lumbar spine or hip region (Tables 2 and 3).

The iPTH levels were negatively correlated with BMI and serum 25 (OH) D levels in patients from Changsha and New Brunswick (Table 4, *p* < 0.05). iPTH levels were positively correlated with serum calcium and AKP levels in patients from Changsha and New Brunswick (Table 4, *p* < 0.05). After a multivariate regression analysis, iPTH remained significantly correlated with serum calcium and AKP levels in patients from both cities (*p* < 0.05) (Table 4).

## 4. Discussion

The understanding of PHPT has been changing substantially over the last few decades [2]. By comparing the clinical presentation of PHPT patients from New Brunswick (USA) and Changsha (China) over the same time period, we found that biochemical and clinical differences between the two countries still exist. Our current data showed that PHPT patients from Changsha have higher serum calcium, iPTH, and AKP levels, lower 25 (OH) D levels, and lower bone mineral density, suggesting that they have more severe PHPT at the initial presentation.

The mechanisms of these distinct differences between the two cities remain unknown. One possibility is that PHPT patients in the USA were diagnosed at the asymptomatic stage, since most people in the USA receive annual physical examinations [1, 2]. In China, most PHPT patients were diagnosed when they came to hospital due to kidney stones, skeletal lesions, or other symptoms related to hypercalcemia. The duration of PHPT in China is about one year shorter than that in the USA as reported by Liu et al. [6]. The duration of PHPT from the two cities has no significant difference in our current study. The wide use of multichannel chemistry screening tests in China recently may be responsible for the discrepancy.

Another possibility is the nutritional and environmental differences between New Brunswick and Changsha. The lower 25 (OH) D levels in Chinese patients may reflect low vitamin D intake or less sun exposure [7, 8]. Although serum 25 (OH) D levels are higher (14.2 ng/mL) than those in cohorts from Shanghai (13 ng/mL) and Beijing (8.8 ng/mL), the levels are still much lower than those in patients from New Brunswick (28.0 ng/mL). Our data also show that iPTH levels were much higher and better correlated with calcium in PHPT patients from Changsha. It is consistent with previous studies showing that lower 25 (OH) D levels and higher iPTH levels are usually associated with more severe PHPT profiles [9, 10]. Higher iPTH might also inhibit liver vitamin D-binding protein production and further lower total 25 (OH) D levels [11]. We hypothesize that long-term low 25 (OH) D levels might induce parathyroid gland hyperplasia and subsequent development of adenoma. A previous study found a significant inverse relationship between serum 25 (OH) D and gland weight, which indicated that lower 25 (OH) D levels are usually associated with heavier parathyroid gland weight, usually leading to severe PHPT [12]. Saliba et al. reported that the sufficient threshold of the prevention of secondary hyperparathyroidism in persons with normal renal function is 25 (OH) D > 20 ng/mL [13]. Long-term vitamin D deficiency can also mask hypercalcemia and delay the diagnosis of PHPT. It is reasonable to measure 25 (OH) D levels and treat low 25 (OH) D levels in all patients with elevated PTH level [14].

We also find a difference in the pathology between these two groups of PHPT patients for the first time. Around 92% of PHPT patients who had surgery in Changsha were found to have parathyroid adenoma, which is twice as many as those in New Brunswick (*p* < 0.01). Most of the PHPT patients in the US are postmenopausal women, and US PHPT patients are also heavier with higher fasting glucose levels, which may also reflect different genetic backgrounds between the two groups of patients as well as different genetically determined PTH metabolism [15]. The higher percentage of parathyroid hyperplasia might be due to selection bias. This study cannot therefore be generalized for all PHPT patients in New Jersey or US. Further studies including better classifications, specific biochemical marker, and transcriptional patterns for adenoma or hyperplasia are needed.

Low bone mineral density and higher AKP levels suggest that Chinese PHPT patients might have more severe bone disease than those in the USA. In our present study, BMD is lower in both the lumbar spine and hip regions, while the previous Shanghai cohort only showed significantly lower BMD in the lumbar spine region [6]. Our data suggest that different living conditions and geographic regions may also affect clinical features of PHPT patients [16]. A greater percentage of male patients with PHPT have more kidney stones than female patients. We suggest that female PHPT patients might be more sensitive to bone loss and that male patients might be more likely to develop renal disease. This finding is consistent with a previous study conducted by De Lucia et al. [17]. The PTH level may also have different influences on bone metabolism and metabolic syndrome among different ethnicities and genders [18].

Another interesting finding is that there are more male and premenopausal female PHPT patients from Changsha than from Western countries. This finding is consistent with previous studies in Chinese and Indian PHPT patients [3, 6, 19]. Our data showed similarities between male and premenopausal female PHPT patients which is consistent with the recent study by Castellano et al. [20]. The PHPT patients from Changsha are also younger at diagnosis with much higher iPTH levels and more renal stones, pointing to a possible genetic impact on the presentation of PHPT. Compared to Caucasian and Asian PHPT patients, African American patients presented a more severe PHPT profile, which supports this point [4].

The potential limitation of the current study is that all patients are from two regional referral centers in China and the USA, leading to possible selection bias. We do not have X-ray, ultrasound, or CT scans for asymptomatic kidney stones, so the true prevalence of kidney stones may be different.

## 5. Conclusions

PHPT patients from Changsha present with more severe PHPT profiles and more kidney stones at their initial evaluation. A further study in a larger cohort is warranted to confirm our findings and to explore the underlying mechanisms.

## Figures and Tables

**Table 1:** Comparison of clinical features of PHPT patients in New Brunswick and Changsha from 2010 to 2016.

Clinical features	Changsha		New Brunswick		*p* value
	*n* = 131	Normal range	*n* = 169	Normal range	
Age (years)	52.4 ± 15.4		58.4 ± 12.5		<0.01
Female (% )	66% (87/131)		77% (130/169)		<0.05
Duration (years)	5.8 ± 7.7		4.4 ± 6.4		0.7
BMI (kg/m^2^)	21.5 ± 3.9		29.3 ± 6.5		<0.001
Ca (mg/dL)	13.1 ± 2.3	8.4–10.1	11.1 ± 0.6	8.6–10.4	<0.001
Serum PTH (pg/mL)	676.4 ± 564.7	14.0–63.0	138.7 ± 99.7	15.0–65.0	<0.001
25 (OH) D (ng/mL)	14.2 ± 8.1	30.0–100.0	29.5 ± 11.0	30.0–80.0	<0.001
Albumin (g/dL)	3.9 ± 0.6	4.0–5.5	4.3 ± 0.5	3.5–5.5	<0.001
Corrected Ca (mg/dL)	13.2 ± 2.5		10.8 ± 0.7		<0.001
AKP (U/L)	383.7 ± 645.9	50.0–135.0	86.0 ± 30.4	45.0–115.0	<0.001
Glucose (mg/dL)	92.7 ± 23.2	70.0–110.0	99.7 ± 19.6	65.0–99.0	<0.001
Creatinine (mg/dL)	1.1 ± 0.7	0.5–1.5	0.9 ± 0.3	0.5–1.2	0.7
Kidney stone (%)	62% (81/131)		31% (52/169)		<0.001
Adenoma (%)	92% (85/92)		48% (77/159)		<0.001

PTH: parathyroid hormone; BMI: body mass index; Ca: serum calcium; 25 (OH) D: 25-hydroxyvitamin D; AKP: alkaline phosphatase.

**Table 2:** Comparison between male and pre- and postmenopausal female PHPT patients from New Brunswick and Changsha.

Clinical features	Male (*n* = 39) NB	Male (*n* = 44) CS	Pre-F (*n* = 28) NB	Pre-F (*n* = 33) CS	Post-F (*n* = 102) NB	Post-F (*n* = 54) CS
Age (years)	59.1 ± 10.7^a,b,e^	49.3 ± 16.6^c,d^	39.4 ± 8.7^b^	38.7 ± 9.7^d^	63.3 ± 8.6	63.2 ± 7.6
Duration (years)	4.6 ± 6.3	6.7 ± 8.1	3.1 ± 3.2	4.3 ± 6.0	4.7 ± 7.0	6.0 ± 8.2
BMI (kg/m^2^)	30.4 ± 4.9^a,e^	21.7 ± 3.2	27.4 ± 6.7^c^	19.8 ± 4.8^d^	29.3 ± 7.0^d^	22.3 ± 3.8
Serum Ca (mg/dL)	11.2 ± 0.6^e^	13.4 ± 2.2	11.2 ± 0.6^c^	13.4 ± 2.7	11.0 ± 0.5^d^	12.8 ± 2.2
Serum PTH (pg/mL)	157.7 ± 125.0^b,e^	788.4 ± 519.5^d^	170.3 ± 156.5^b.c^	801.9 ± 637.6^d^	122.8 ± 58.5^d^	503.2 ± 516.7
25 (OH) D (ng/mL)	27.7 ± 9.3^e^	14.3 ± 8.7	26.7 ± 12.7^c^	13.9 ± 7.3	30.8 ± 10.9^d^	14.2 ± 8.3
Albumin (g/dL)	4.4 ± 0.6^e^	3.8 ± 0.5	4.4 ± 0.3^c^	4.0 ± 0.5	4.3 ± 0.4^d^	3.8 ± 0.7
AKP (U/L)	88.9 ± 33.2^e^	384.1 ± 564.8^d^	92.3 ± 42.2^c^	794.0 ± 1034.3^d^	83.2 ± 25.1^d^	166.1 ± 234.2
Glucose (mg/dL)	102.8 ± 20.8^e^	94.9 ± 27.1	98.3 ± 21.6	87.5 ± 19.7	98.8 ± 18.5	94.1 ± 21.5
Creatinine (mg/dL)	1.09 ± 0.3^a,b^	1.3 ± 0.8^c,d^	0.6 ± 0.1^b,c^	0.9 ± 0.6	0.8 ± 0.2	0.9 ± 0.4
Kidney stone (%)	44% (17/39)^a,b,e^	77% (34/44)^c,d^	29% (8/28)^c^	52% (17/33)	26% (27/102)^d^	55.6% (30/54)
Total hip BMD (*T*-score)	−0.697 ± 0.927^a,b,e^	−2.532 ± 1.190	−0.167 ± 0.823^b,c^	−2.594 ± 1.557	−1.075 ± 1.100^d^	−2.439 ± 1.060
L1–4 BMD (*T*-score)	−0.295 ± 1.492^b,e^	−2.536 ± 1.497^d^	−0.515 ± 1.203^b,c^	−2.529 ± 1.240^d^	−0.839 ± 1.501^d^	−2.930 ± 0.968

Pre-F: premenopausal women; Post-F: postmenopausal women; NB: New Brunswick; CS: Changsha; BMI: body mass index; AKP: alkaline phosphatase; Ca: calcium; 25 (OH) D: 25-hydroxyvitamin D. Significant level *p* < 0.05. ^a^Significant vs. Pre-F in NB. ^b^Significant vs. Post-F in NB. ^c^Significant vs. Pre-F in CS. ^d^Significant vs. Post-F in CS. ^e^Significant vs. male in CS.

**Table 3:** Comparison of BMD of PHPT patients in New Brunswick and Changsha from 2010 to 2016.

Clinical features	Changsha (*n* = 131)	New Brunswick (*n* = 169)	*p* value
Total hip BMD (g/cm^2^)	0.623 ± 0.150	0.900 ± 0.150	<0.001
Total hip BMD (*T*-score)	−2.507 ± 1.212	−0.839 ± 1.070	<0.001
Total hip BMD (*Z*-score)	−1.572 ± 1.486	−0.195 ± 0.978	<0.001
L1–4 BMD (g/cm^2^)	0.713 ± 0.149	1.077 ± 0.195	<0.001
L1–4 BMD (*T*-score)	−2.691 ± 1.256	−0.659 ± 1.466	<0.001
L1–4 BMD (*Z*-score)	−1.608 ± 1.532	0.127 ± 1.434	<0.001

BMD: bone mineral density.

**Table 4:** Simple linear regression and multiple linear regression of relation adjusted for age and BMI (partial correlation coefficients) between serum iPTH and other variables in patients with primary hyperparathyroidism in Changsha and New Brunswick and all patients in the two cities.

Serum PTH (pg/mL)	Simple linear regression			Multiple linear regression		
	Changsha (*n* = 131)	New Brunswick (*n* = 169)	Total (*n* = 300)	Changsha (*n* = 131)	New Brunswick (*n* = 169)	Total (*n* = 300)
Age (years)	−0.29^b^	0.03	−0.21^b^	−0.01	0.04	−0.04
Duration (years)	0.03	−0.03	0.01	0.08	−0.01	0.01
BMI (kg/m^2^)	−0.28^b^	0.02	−0.45^a^	−0.14	0.01	−0.06
Ca (mg/dL)	0.43^a^	0.13^c^	0.54^a^	0.44^a^	0.23^b^	0.50^a^
25 (OH) D (ng/mL)	−0.25^c^	−0.20^c^	−0.53^a^	−0.15	−0.14	−0.11^c^
AKP (U/L)	0.69^a^	0.23^b^	0.60^a^	0.59^a^	0.34^a^	0.63^a^
Cr (mg/dL)	0.22^c^	0.01	0.11	0.11	0.01	0.08

PTH: parathyroid hormone; BMI: body mass index; Ca: serum calcium; Cr: creatinine; 25 (OH) D: 25-hydroxyvitamin D; AKP: alkaline phosphatase. Data are presented as *r*: ^a^*p* < 0.0001, ^b^*p* < 0.01, and ^c^*p* < 0.05.

## Data Availability

The data used to support the findings of this study are available from the corresponding author upon request.
